# CYP2D6 Pharmacogenetics in Nigerian Sickle Cell Disease: Phase 1 of Implementing Pharmacogenomics Testing for Effective Care and Treatment in Africa (iPROTECTA) program

**DOI:** 10.12688/gatesopenres.16370.1

**Published:** 2025-11-10

**Authors:** Babatunde Adeagbo, Olusola Olarewaju, Ochuko Orherhe, Zedias Chikwambi, Adrian Mazhindu, Rahman Bolarinwa, Oluseye Bolaji, Collen Masimirembwa, Consortium for Genomics and Therapeutics in Africa

**Affiliations:** 1 Obafemi Awolowo University Faculty of Pharmacy, Ife, Osun, 220282, Nigeria; 2 Obafemi Awolowo University Teaching Hospital Complex, Ile-Ife, Osun, 220282, Nigeria; 3 African Institute of Biomedical Science and Technology, Harare, Harare South, Zimbabwe; 4 Obafemi Awolowo University Faculty of Basic Medical Sciences, Ile-Ife, Osun, 220282, Nigeria

**Keywords:** Sickle Cell Disease, pre-emptive pharmacogenomic, CYP2D6, phenotype, genotype, opioids

## Abstract

**Background:**

Sickle Cell Disease (SCD) is highly prevalent in Nigeria, with severe pain crises being a primary cause of morbidity. Codeine and tramadol are frequently used opioids, but their effectiveness and safety are significantly influenced by *CYP2D6* genetic variations. Clinical Pharmacogenetics Implementation Consortium (CPIC) guidelines exist for opioid therapy based on CYP2D6 phenotypes. There’s a critical need for pre-emptive pharmacogenomic (PGx) testing in African SCD patients to guide opioid selection. This study aimed to determine *CYP2D6* allele, phenotype frequencies and evaluate the feasibility of implementing pre-emptive pharmacogenomic (PGx) testing to guide opioid therapy for SCD patients in Nigeria.

**Methods:**

This prospective, multicenter implementation study recruited 503 consenting SCD patients (HbSS or HbSC) aged ≥15 years from five Nigerian sites. Blood samples were collected for DNA extraction. *CYP2D6* single-nucleotide polymorphisms and copy number variations were determined using Taqman assays based open array, GenoPharm. Phenotypes were assigned based on Clinical Pharmacogenetics Implementation Consortium (CPIC) guidelines using the Genomics Information Management System (GIMS). and patient-specific medication safety cards were generated.

**Results:**

We successfully genotyped 503 SCD patients with a mean age of 25.1 years, while 61.4% were female, and hydroxyurea use was less than 9.4%. Actionable *CYP2D6* variants were found in 36.6% of participants. The predicted phenotype distribution was 8.8% Ultrarapid Metabolizers (UM), 54.1% Normal Metabolizers (NM), 26.0% Intermediate Metabolizers (IM), and 1.8% Poor Metabolizers (PM), with 9.3% undetermined. Patient medication safety cards were provided to guide prescriptions.

**Conclusions:**

This study successfully established a genotyped cohort of 503 Nigerian SCD patients, demonstrating the feasibility of pre-emptive pharmacogenetic testing through a Pan-African collaborative model in a resource-limited setting. The identification of PM and UM provides direct clinical guidance, as CPIC guidelines recommend avoiding codeine and tramadol in these groups due to the high risk of diminished efficacy or serious toxicity, respectively. The high prevalence of actionable CYP2D6 variants indicates a substantial proportion of Nigerian SCD patients may experience altered opioid responses, underscoring the need for tailored prescribing to optimise pain control and minimise adverse drug reactions.

## Introduction

Sickle cell disease (SCD) is the most common hemoglobin disorder worldwide, with the highest prevalence in sub-Saharan Africa, afflicting 2-3% of all Nigerians ^1, 2^ SCD is an inherited haemoglobinopathy resulting from a base substitution of valine for glutamic acid at position 6 of the beta-globin gene, which encodes an abnormal hemoglobin S. ^3, 4^ SCD presents a considerable health challenge in Nigeria and Africa at large, with vaso-occlusive crises (VOC) being the most frequent and debilitating complication, though precise per-patient incidence data is limited. ^5, 6^ Studies in Nigeria reveal high rates of VOC among SCD patients, often leading to hospital admissions, with children with SCD constituting a notable percentage of overall hospitalizations in certain facilities. ^5– 8^ While comprehensive statistics on SCD-related hospital admissions across Africa is not readily available due to data collection challenges, existing hospital-based research underscores the significant contribution of SCD to both pediatric and adult hospital stays, primarily driven by pain crises and associated complications. ^9^ This significantly impacts school attendance and academic performance among SCD children in Africa, particularly in Nigeria. ^10, 11^ In Nigeria, studies reveal that adolescents with SCD, aged 12 to 18, miss an average of 12% of the school year, with over 35% missing at least one month. ^11^ A comparison of children with SCD and their siblings in Nigeria found a significantly higher school absence rate in the SCD group, alongside a greater percentage of below-average students, indicating a direct correlation between disease severity and educational disruption. ^12^ Similar trends have been observed across Sub-Saharan Africa with frequent hospital visits and prolonged crisis episodes leading to extended periods of school absenteeism, highlighting the widespread educational burden of VOCs in this vulnerable population. ^11, 13^

While effective pain management aims for rapid resolution, the current average time to fully resolve a VOC pain in patients can still be prolonged, often ranging from several days to over a week, depending on the severity and individual response. ^14, 15^ Incremental and combination therapy have been shown to significantly shorten this duration. ^16^ For instance, studies have shown that a multimodal approach combining opioids (like codeine or tramadol) with paracetamol and NSAIDs, when initiated promptly and titrated appropriately, can lead to substantial pain reduction within 24-48 hours, with many patients achieving satisfactory pain control and functional improvement within 3-5 days, compared to longer durations with less comprehensive approaches. ^15, 17– 19^

Inter-individual variation in pain severity, perception, experiences and responses, including toxicity to opioids, has been observed and partly attributed to genetic polymorphism of opioid receptors and metabolic enzymes. ^20^ In pain management, Pharmacogenomics (PGx) can play a significant role in optimizing symptom control and adverse drug effects. Opioids are metabolized to a larger extent via the cytochrome P450 (CYP) system, particularly isoenzymes CYP2D6, CYP2C9, CYP3A4/5 and uridine 5’-diphosphate-glucuronosyltransferase (UGT). ^21^ The effects of CYP2D6 polymorphisms have been observed, with a high level of evidence, to result in significant differences in response and toxicity in pain management with codeine and tramadol. ^22^ CYP2D6 metabolizes opioids like codeine, oxycodone, hydrocodone, and tramadol into active metabolites that have about 30-fold higher affinity for the mu opioid receptors than the parent molecule. ^23, 24^ CYP2D6 is highly polymorphic, and the key allele with altered activity shows significant geographical and ethnic distribution globally. ^25– 27^ The variants result in different phenotypes. The Clinical Pharmacogenetics Implementation Consortium (CPIC) has categorized these into four CYP2D6 phenotypes—Ultrarapid Metabolizer (UM), Normal Metabolizer (NM), Intermediate Metabolizer (IM), and Poor Metabolizer (PM)—to guide codeine and tramadol therapy. ^28^ For codeine and tramadol, UMs face increased toxicity risk due to rapid conversion to active metabolites, while PMs and often IMs experience reduced analgesic effects due to insufficient conversion; NMs are expected to have a normal response. Consequently, CPIC guidelines recommend avoiding codeine and tramadol in UMs and PMs, using standard dosing for NMs, and considering alternatives or cautious use with monitoring for IMs, all to optimize efficacy and minimize adverse drug reactions. ^29, 30^

Clinical implementation of CYP2D6 pharmacogenomic-biomarker-guided pain management is needed, given the burden of patients with SCD-associated pain crises. Pre-emptive pharmacogenomic-biomarker testing to guide opioid use in SCD patients in an African setting has so far not been implemented to the best of our knowledge. Targeting specific at-risk populations for acute pain episodes like SCD patients for pre-emptive CYP2D6 genotyping for guided pain intervention is the future that could have enormous benefits by minimizing pain impact on health-related quality of life (QoL), in-hospital periods, reducing cost of care, potentially improving prognosis and enhancing confidence in the use of codeine among pediatric SCD patients. ^31^

Successful implementation of pre-emptive pharmacogenomic-biomarker guided treatment has setting-specific challenges, including limited PGx knowledge among clinicians, lack of equipment, limited evidence generated among populations in Africa and importantly, laboratory-clinic integration. This study aims to tackle these challenges by amplifying the reach of currently available resources on the African continent and setting up a much-needed clinical implementation project that demonstrates this benefit in outcomes for SCD patients and probes the effectiveness of this approach. The primary objective of this clinical implementation study is to evaluate the feasibility and effectiveness of opioid selection based on CPIC recommendations in SCD patients in Nigeria using pre-emptive testing. Furthermore, the study aims to generate more clinical, metabolic/pharmacokinetic data on opioid treatment outcomes among Africans. This paper presents the first phase of pre-emptive CYP2D6 genotyping in Nigerian SCD patients.

## Methodology

**Figure 1.**
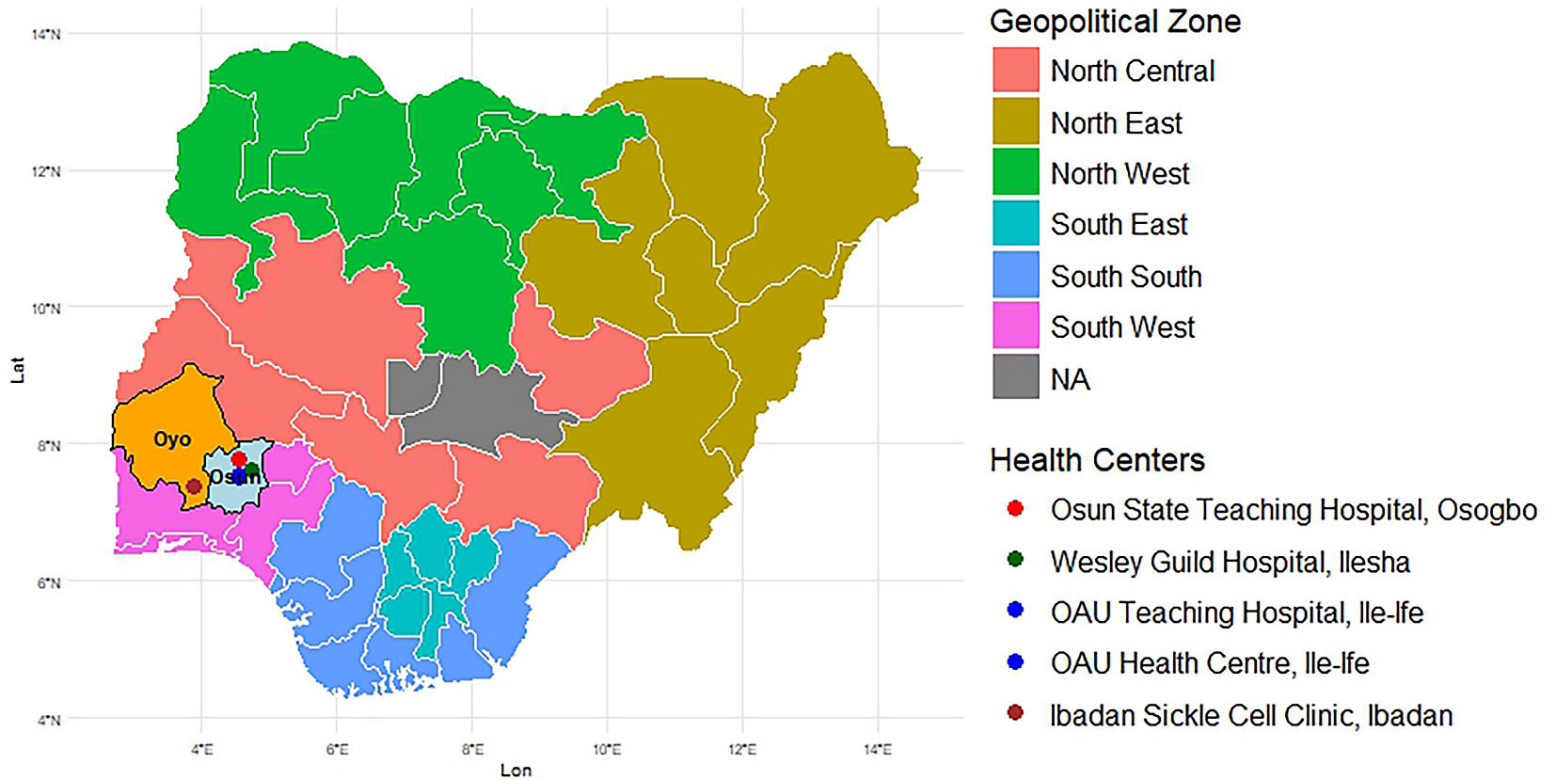
The locations of the study sites in Nigeria.

This was a prospective, multicenter implementation study of pharmacogenomics-guided opioid treatment for SCD-associated pain crises in Nigeria. It was conducted at five sites in Nigeria: Obafemi Awolowo University (OAU) Teaching Hospitals Complex (OAUTHC), Adult and Pediatric Hematology clinics, OAUTHC Wesley Guild Hospital Unit, OAU Health Centre, Osun State Teaching Hospital (OSTH), and Ibadan Sickle Cell Foundation Clinic (ISCF), Ibadan, Oyo State ( Figure 1).

The study was performed following the Declaration of Helsinki and the relevant regulations and laws governing research in Nigeria. Ethical approval was obtained from the Obafemi Awolowo University Teaching Hospitals Complex Research Committee (ethical clearance number ERC/2022/07/17), and this was domesticated in all the other sites outside OAUTHC.

Written informed consent was obtained from all participants in a language the participant understood, which was English or the local language, mainly Yoruba and Igbo. Participants were thoroughly informed about the study’s purpose, procedures, potential risks and benefits, their right to withdraw at any time without penalty, and the confidentiality of their data. Ample opportunity was provided for participants to ask questions, and all queries were addressed to their satisfaction before consent was obtained. For participants who were minors (less than 18 years of age), written informed consent was obtained from their parents or legal guardians. In addition, written assent was obtained directly from the minors themselves, ensuring they understood the study in an age-appropriate manner and willingly agreed to participate. The assent process involved explaining the study in simple, clear language, addressing any concerns they might have, and emphasizing the voluntary nature of their participation. No minor was included in the study without both parental/guardian consent and their assent. All informed consent and assent procedures adhered strictly to the guidelines and requirements of the Obafemi Awolowo University Teaching Hospitals Complex Ethics and Research Committee.

In the study, 503 consenting participants who had been diagnosed with HbSS or HbSC using hemoglobin electrophoresis were recruited. After informed consent, each of the subjects was screened for eligibility, and those who met the eligibility criteria were recruited, and data were entered into the database. Inclusion criteria for the study were participants above 15 years of age and with estimated glomerular filtration rate (eGFR) ≥ 60mL/min/1.73/m2. Exclusion criteria for the study were pregnancy, breastfeeding and previous experience of bone marrow transplantation, history of previous side effects to opioids and derangement in liver enzymes.

**Figure 2.**
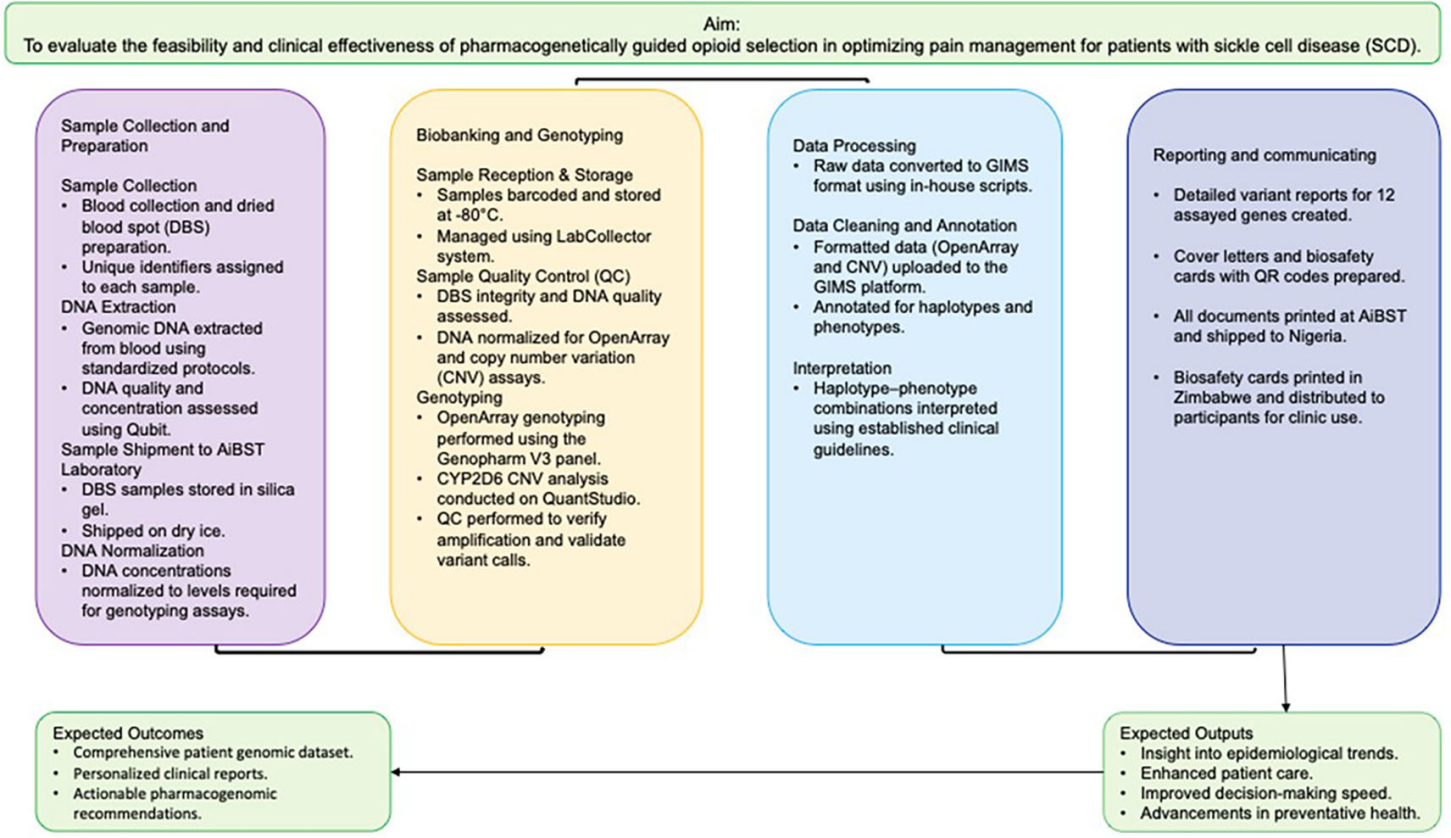
The workflow chart for the genotyping.

Blood samples were collected into 5 mL EDTA tubes from each of the consented participants. Blood aliquots of 200 μl and dry blood spot paper were made and stored at -80°C until DNA extraction. Genomic DNA (gDNA) was isolated in Nigeria from whole blood samples using QIAamp DNA Blood Mini Kit (Qiagen, Hilden, Germany), following the manufacturer’s instructions. The concentration and quality of the extracted gDNA were assessed using a spectrophotometer (e.g., NanoDrop One, Thermo Fisher Scientific). The samples were shipped to the African Institute of Biomedical Science and Technology (AiBST), Harare, Zimbabwe, on an ice pack, as DNA and dried blood spots. The sample processing workflow is outlined in Figure 2. All received samples were verified (labelling, sample type), registered and bio-banked at -80°C using the Lab Collector laboratory information management system (LIMS) Biobanking module at AiBST. DNA Concentration was determined using Qubit 3 (Thermo Fisher Scientific, Waltham, Massachusetts, USA). Samples that had a DNA concentration less than 5 ng/μl, DNA were re-extracted from DBS. This procedure was done to ensure adequate DNA concentration for further analysis.

The genotyping was done using GenoPharm ^®^ custom open array platform (Thermo Fisher Scientific Open Array™ system), which evaluates 120 Single Nucleotide Polymorphisms (SNPs) across 46 pharmacogenes, specifically designed to be inclusive of variants common in African populations. ^32^ The full list of pharmacogenes and specific target variants included in this panel is provided is as reported by Kanji CR et al. 2023. ^32^ For the single-nucleotide variations, the process utilized TaqMan Assay chemistry with polymerase chain reaction (PCR) on the Applied Biosystems QuantStudio 12K Flex Real-Time PCR System. Genomic DNA samples were diluted to 50 ng/μL, mixed with TaqMan Genotyping master mix, and transferred to the open array via an automated system. Genotypes were determined using TaqMan Genotyper Software. For Copy Number Variation (CNV) determination, for CYP2D6, the Applied Biosystems TaqMan copy number assays targeting Exon 9 were used. This involved a duplex real-time PCR reaction with the human TaqMan Copy number reference assay RnaseP serving as the reference gene. To ensure the accuracy and reliability of the reported genotypes, robust quality control measures were implemented. All genotyping data were analyzed using TaqMan™ Genotyper Software, requiring a confidence value of ≥ 0.95 for a call to be assigned. Data points below this threshold were classified as undetermined. The GenoPharm ^®^ platform was validated as previously described. ^32^ For genotype validation, a subset of 5% of the cohort’s samples was randomly selected. These samples were cross-validated for the most clinically actionable variants (specifically the *CYP2D6 *4, *10, 17, and 29 alleles). The relative quantification of CYP2D6 copy numbers was performed using Copy Caller Software v2.1. Copy number was predicted based on the comparative cycle threshold (CT) between the sample and RnaseP, leading to a ΔΔCT calculation, with successful calls requiring a confidence value greater than 0.95 and a z-score less than 1.75. Genotype distributions were tested for compliance with Hardy–Weinberg equilibrium (HWE) by calculating the expected frequencies of genotypes and comparing them to the observed values using the chi-squared (χ ^2^) test. Categorical data were summarized using proportions and percentages. The CNV and SNP genotyping results were transformed into the GIMS input format using a custom R script. The GIMS ready sample files were then uploaded onto the GIMS platform according to the Biologis standard operating procedure. The output files consisted of three PDF files: a cover letter, a Safety Card, and a main detailed report. The Patient Medication Safety Card was then printed, and the username and password were provided separately for each patient.

## Results

### Patient recruitment

A total of 503 SCD patients, who consented to the study and biobanking across borders, were recruited over 8 months. Table 1 provides a summary of the demographic characteristics of the 503 patients enrolled across the six study sites. The mean age of the patient cohort was 25.1 years, with ages ranging from 15 to 75 years. A majority of the patients were female, accounting for 61.4% of the total. Regarding BMI categories, 43.1% of the patients had a normal BMI (18.5-24.0), while 33.2% were underweight (<18.5). The most prevalent SCD diagnosis was HBSS, affecting 90.7% of the patients. Ethno-tribal group analysis showed that the Yoruba group constituted the largest proportion at 92.8%. Furthermore, a significant majority of patients (90.5%) were not using hydroxyurea at the time of recruitment.

**Table 1.** Demographics of patients across the sites.

Characteristic	Description	Frequency (%) N = 503	OAUTHC, Adult Clinic	OAUTHC, Pediatrics Clinic	Wesley Hospital Guild	OAU Health Centre	OSTH, Osogbo.	ISCF, Ibadan
Age	Mean (Range)	25.1 (15-75)	28.3 (15-65)	15.2 (15-18)	15.8 (15-30)	23.6 (17-52)	28.1 (16-75)	24.4 (16-59)
Sex								
	Female	61.4	103	5	21	30	40	110
	Male	38.6	55	5	17	17	32	68
BMI category								
	Underweight <18.5	33.2	36	6	27	3	11	84
	Normal 18.5-24.0	43.1	72	3	9	33	41	59
	Overweight 25-29.9	15.3	30	1	2	9	17	18
	Overweight 30-34.9	8.4	20	0	0	2	3	17
SCD diagnosis								
	HBSC	9.3	25	1	3	0	10	8
	HBSS	90.7	133	9	35	47	62	170
Ethno-tribal group								
	Yoruba	92.8	148	10	36	39	68	166
	Igbo	6.4	9	0	1	8	4	10
	Hausa	0.4	0	0	1	0	0	1
	Minor	0.4	1	0	0	0	0	1
Hydroxyurea usage at recruitment								
	Yes	9.5	12	7	19	0	1	9
	No	90.5	146	3	19	47	71	169

### Patient genotyping

**Figure 3.**
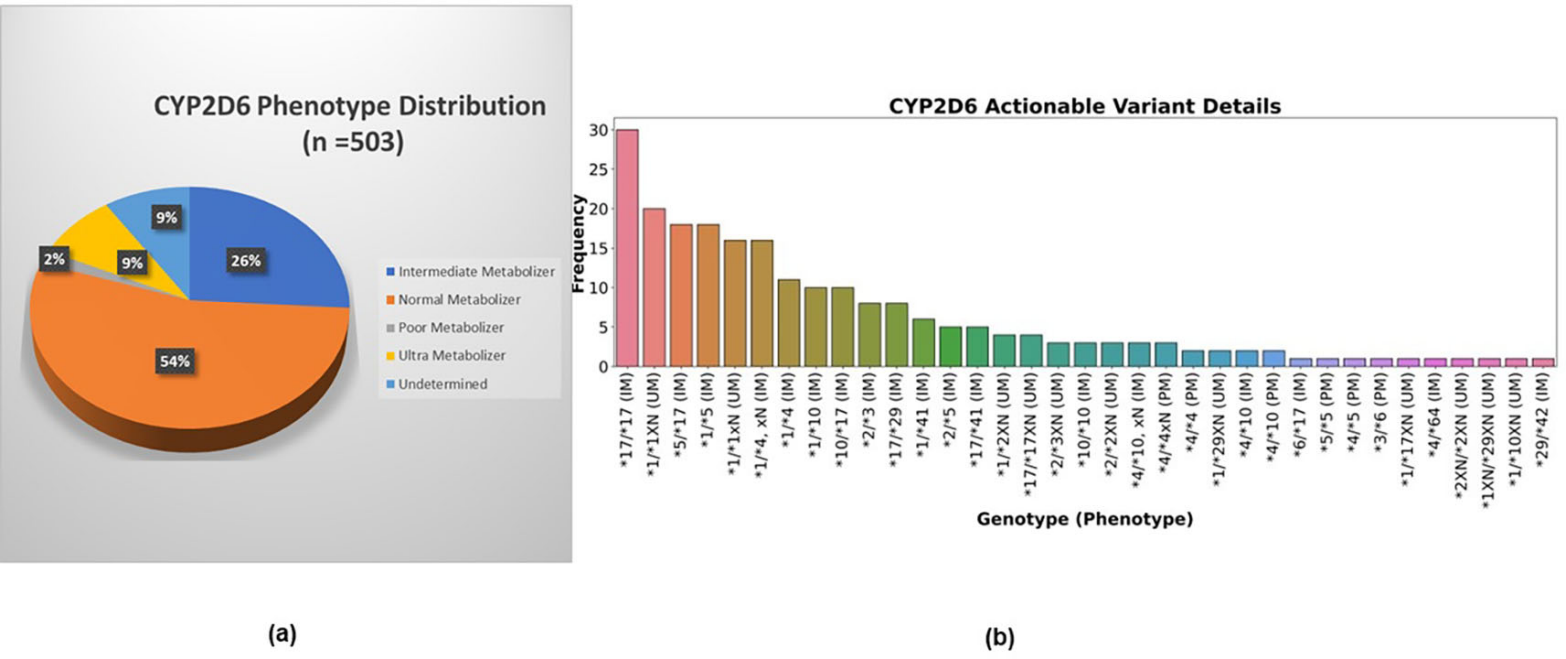
CYP2D6 variants distribution (a) Phenotype distribution (b) Actionable variants genotype distribution (n = 503).

The dried blood (DBS) and the DNA samples for each patient were successfully shipped within 4 to 7 days to Zimbabwe in three batches. Some of the DNA samples that had issues were re-extracted using the DBS supplied. The turnaround of blood sample collection, shipping samples from Nigeria to Zimbabwe and return of results varied greatly from 1 to 3 months in this pilot study. Table 2 presents the details of the distribution of CYP2D6 single-nucleotide variants (SNVs) across six study sites in Nigeria for the SCD patients. As shown in Table 1 different CYP2D6 SNV genotypes were found within the population. The most frequently observed genotype was *1/*1, accounting for 24.5% (n = 123) of the total, followed by *1/*17 at 17.7% (n = 89). Other notable genotypes included *1/*1XN (5.2%, n = 26), *1/*2 (3.6%, n = 18), and *1/*4 (2.2%, n = 11). Several SNVs were present at lower frequencies, such as *1/*10 (2.8%), *1/*29 (2.0%), and *1/*5 (2.8%). A substantial portion of samples, 9.3% (n = 47), were categorized as “UND” (undetermined) because their genotypes could not be determined. The distribution of these SNVs varied across the individual study sites. Table 3 and Figure 3 further describe the distribution of CYP2D6 actionable variants, categorized by their corresponding phenotypes, across the six study sites. Of the phenotypes observed, the Intermediate Metabolizer (IM) phenotype was the most prevalent, accounting for 26.0% (n = 131) of the total. This phenotype is characterized by the presence of one decreased function and one functional allele, with genotype definitions including combinations such as *1/*4, *1/*41, *10/*17, and *17/*17. The Ultrarapid Metabolizer (UM) phenotype, defined by the carrier of duplications of functional alleles, was observed in 8.7% (n = 44) of the patients, with genotype definitions like *1/*1XN and *17/*17xN. The Poor Metabolizer (PM) phenotype, characterized by two non-functional alleles (e.g., *4/*4, *5/*5), was the least frequent, representing 1.8% (n = 9) of the cohort. These phenotypes are driven by the variants CYP2D6 *4, *5, *10, *17, and *41. The top 3 genotypes were *17/*17, *1/*4xN, and *1/*1xN as shown in Figure 3.

**Table 2.** Distribution of the CYP2D6 SNVs across study sites in Nigeria.

SNVs	OAUTHC	OAUTHC-P	OAU-HC	WGH	OSTH	IBD -SCF	Total	% Frequency
*1/*1	40	3	10	7	18	45	123	24.5
*1/*10	4	1	2		4	3	14	2.8
*1/*10, xN	0	1		1	1	1	4	0.8
*1/*17	24	1	8	8	17	31	89	17.7
*1/*17XN						1	1	0.2
*1/*1XN	5	1	4	1	3	12	26	5.2
*1/*2	6		2	2	3	5	18	3.6
*1/*2XN	2		1			1	4	0.8
*1/*29	1	1	2	4	1	1	10	2
*1/*29XN	1				1		2	0.4
*1/*4	5		1	1		4	11	2.2
*1/*4, xN	4			3	2	7	16	3.2
*1/*41	2					4	6	1.2
*1/*41XN	0		1			1	2	0.4
*1/*5	2		1	2	3	6	14	2.8
*10/*17	6					4	10	2
*10/*17XN	0					2	2	0.4
*17/*17	9	2	3	1	5	10	30	6
*17/*17, xN	4					1	5	1
*17/*41	0				1	3	4	0.8
*17/*29	4		3			1	8	1.6
*1XN/*29XN					1		1	0.2
*2/*2	1						1	0.2
*2/*2XN	1						1	0.2
*2/*29	4		1		2	3	10	2
*2/*3	3		1	1		2	7	1.4
*2/*3XN	2						2	0.4
*2/*5	2				1	2	5	1
*2XN/*2XN			1				1	0.2
*29/*42				1			1	0.2
*3/*6						1	1	0.2
*4/*10	1					1	2	0.4
*4/*10, xN	0		1			3	4	0.8
*4/*4, xN					1	2	3	0.6
*4/*4	0			1		1	2	0.4
*4/*5						1	1	0.2
*4/*64						1	1	0.2
*6/*17						1	1	0.2
*5/*5						1	1	0.2
*5/*17	1		1		3	4	9	1.8
*10/*10	2			1			3	0.6
UND	22		4	4	5	12	47	9.3
Total	158	10	47	38	72	178	503	100

**Table 3.** The distribution of the haplotype and phenotype of CYP2D6 actionable variants across study sites.

Haplotype	Actionable phenotype	Genotype definition	Counts of actionable phenotypes across sites						Total count	Total frequency (%) phenotype
			OAUTHC	OAUTHC-P	OAU-HC	WGH	OSTH	IBD -SCF		
*1/*4 (n=?); *1/*4 (n=?), xN; *1/*41; *1/*5; *10/*10; *10/*17; *17/*17; *17/*29; *17/*41; *2/*3; *2/*5; *29/*42; *4/*10; *4/*10, xN; *4/*64; *5/*17; *6/*17	IM	One decreased function and one functional allele	42	2	12	10	16	49	131	26.0
*3/*6; *4/*4; *4/*4xN; *4/*5; *5/*5; *4/*10	PM	Two non-functional alleles	0	0	1	1	1	6	9	1.8
*1/*1xN; *1/*17xN; *1/*10xN; *17/*17xN; *1xN/*29xN; *1/*29xN; *1/*2xN; *2xN/*2xN; *2/*3xN	UM	Carrier of duplications of functional alleles	15	1	6	2	5	15	44	8.8

### Patient medication safety cards

A patient medication safety card was printed and given to each of the 503 SCD patients ( Figure 3). The card is meant for presentation at every clinical day before consultation in order to guide prescription. For each of the patients with phenotype results, detailed genotype reports and a recommended treatment plan report were provided for the primary physician of each patient. The study clinicians now have a database of the genotypes of their patients in preparation for Phase 2 of the study, when this information will be used to select drugs and doses when the patients present with pain episodes.

## Discussion

SCD-associated VOCs are characterized by intense pain, often requiring hospitalization and medical interventions, which can result in missed school days or workdays, low economic productivity and poor quality of life ^10, 11^ (QoL). With SCD disease occurring at a prevalence of 2-3% in Nigeria, and patients having up to 6 VOC episodes a year, ^2, 33, 34^ need to effectively and efficiently manage the pain episodes is of public health importance. One of the ways of achieving this is the possibility of using a precision medicine approach in the use of codeine and tramadol. In this two-phase study, we aimed to pre-emptively genotype SCD patients at selected hospitals during phase 1 and then use that genetic information to inform drug choice and dose optimization in treating the patients in phase 2.

This study represents Phase I of the Implementing Pharmacogenomics Testing for Effective Care and Treatment in Africa (iPROTECTA) program to evaluate the feasibility of implementing pre-emptive pharmacogenetic testing in pain management in SCD patients in Nigeria. In this phase, we successfully identified 5 hospitals that run SCD treatment clinics and recruited 503 SCD patients. The most commonly used opioids in pain management include codeine, tramadol and morphine. The patients were genotyped for CYP2D6 variants important in the safe use of codeine and tramadol, in pain management in SCD patients.

This study successfully established a pre-emptively genotyped cohort of 503 Sickle Cell Disease patients in Nigeria, with samples shipped to Zimbabwe for analysis within 4-7 days and results returned in approximately three months. The first of three batches of Dried Blood Spot (DBS) samples encountered issues, a common challenge in transnational biobanking. Literature on establishing biobanks and genomic studies in Africa frequently highlights logistical complexities, including sample degradation due to variable shipping conditions, customs delays, and insufficient cold chain infrastructure. For instance, studies by Tindana et al. (2016) and Mayne et al. (2017) discussed the multifaceted challenges of biobanking in Africa, emphasizing the need for robust standard operating procedures and contingency planning. ^35– 37^ The project’s proactive mitigation strategy, by subsequently shipping extracted DNA alongside DBS cards, proved crucial in safeguarding sample integrity and ensuring the project’s continued success. This dual-sample approach is a practical solution often recommended to de-risk projects reliant on precious biological materials transported over long distances. ^36^ A crucial element of this success was the thorough training of medical and laboratory personnel in workflow, sample handling, and results interpretation, which significantly enhanced data quality and demonstrated the feasibility of conducting complex genomic studies in resource-limited African settings.

The mean age of our participants was 25.1 years, with a majority of females (61.4%), a figure consistent with findings other studies in the Nigerian or sub-Saharan African population. ^38, 39^ This underscores the severe reality of SCD in Nigeria, where life expectancy remains significantly curtailed, often falling below 50 years despite the increase in awareness and improvement in care management. ^39^ This demographic picture highlights the urgent need for interventions that improve survival and quality of life, in which management of the pain is key in improving the quality of life and decreasing the co-morbidities. ^39^ A significant proportion of our patients are underweight (33.2%) or overweight/obese (23.7%) individuals. This is critical for understanding the manifestation and management of SCD and its associated pain. Body Mass Index (BMI) is increasingly understood to influence SCD outcomes; for example, underweight patients may be at higher risk for certain complications, while obesity can exacerbate inflammation and vascular issues, potentially increasing pain frequency or severity. Studies show that a substantial proportion of children with sickle cell anemia are underweight, and poor growth and nutrition are common in this population due to increased metabolic rates and reduced absorption. ^40– 42^ Thus, in low-resource settings, underweight children with SCD face an increased risk of early mortality. ^41^ The younger age and significantly lower BMI in the Nigerian SCD cohorts when compared to high-income countries suggest a more severe disease course and higher mortality rates in Africa, likely exacerbated by widespread malnutrition. ^40^ This distinct demographic and physiological profile imply that PGx-guided interventions must consider the unique metabolic characteristics of a predominantly younger, often malnourished, patient population. This could influence drug pharmacokinetics and response, potentially necessitating dosage adjustments or alternative drug choices, as drug metabolism and response can be significantly altered by nutritional status and age.

One of the most striking findings in this study is the exceptionally low rate of hydroxyurea (HU) usage, with fewer than 9.5% of participants actively taking this vital, disease-modifying therapy. Participants identified cost as the primary barrier, a finding consistent with numerous reports highlighting financial constraints, limited availability, and insufficient patient/provider education as key obstacles to HU uptake in Nigeria. ^43– 46^ Given HU’s proven efficacy in reducing SCD complications, this widespread underutilization represents a major public health challenge and a significant missed opportunity to improve patient outcomes. Addressing this requires a multi-pronged strategy focusing on affordability, accessibility, and education.

The reported three-month turnaround time (TAT) for genotyping results for 503 patients is considerably longer than typical clinical laboratory TATs for CYP2D6 genotyping, which usually range from 5-10 days in within-country services, with comprehensive panels often taking 7-10 business days. ^47– 49^ The discrepancy between the study’s TAT and standard clinical times is multifactorial, stemming from technical complexities like re-extraction of DNA and quality control. While some laboratories can achieve quick TATs for whole-exome sequencing (2-3 weeks), more complex assays like quantitative PCR for large deletion/duplication testing can take 7-10 weeks ^50^ making the final results for CYP2D6 genotyping take close to the 3 months reported in our project. The observed three-month TAT, though a practical reality for large-scale international research with international sample transfer in a resource-limited setting, highlights a critical operational gap for real-time pharmacogenomics-guided treatment, especially in acute pain management, where timely intervention is crucial. This makes the pre-emptive approach, where at-risk patients have their genotypes already available in local databases, more realistic than reactive testing to address a patient experiencing a pain crisis.

The genotyping results show a substantial proportion of patients with actionable CYP2D6 phenotypes, indicating a significant clinical utility for PGx-guided treatment in this cohort. The study identified 41 different CYP2D6 SNV genotypes. The findings regarding CYP2D6 phenotype prevalence in this study are largely consistent with, yet provide more precise data than, existing literature on Nigerian populations and other African and Asian descent. Previous studies on Nigerian populations report poor metabolizers (PMs) at 2.3% in Yoruba Nigerians and 3.5% across Hausa, Ibo, and Yoruba populations, comparable to rates in other Black (1-4%) and Asian (1-2%) populations. ^51, 52^ This PM frequency contrasts sharply with the higher rates reported for Caucasian populations, which typically range from 5-10%. ^53, 54^ The PM rate of 1.8% in the current study falls within the previously reported range for Nigerians, while the IM rate of 26.0% is notably higher than the 17.4% previously observed. A significant finding from this cohort is the 8.8 % ultrarapid metabolizer (UM) rate, which fills a knowledge gap, as earlier Nigerian studies often did not explicitly detail UM prevalence, ^51^ despite UMs being known to be prevalent in other African populations (e.g., 16% in Ethiopians). ^55, 56^ African populations are characterized by a high prevalence of UMs due to frequent CYP2D6 gene duplications, with rates often exceeding 10% in some ethnic groups. ^57^ The higher IM and identified UM prevalence in the current study provide more comprehensive phenotypic data for a large Nigerian Sickle Cell Disease cohort.

This study revealed a high prevalence of actionable *CYP2D6* variants, present in 36.6% of the genotyped cohort. This underscores the potential value of pharmacogenetic testing in this population, to guide therapy, particularly for pain management. Patients at risk of diminished effectiveness of codeine or tramadol with either IM or PM classification were 27.8% of participants, and those at higher risk of adverse drug effects with UM status were 8.8 %. Given the high risk of VOCs, which can account for as high as 70% of SCD patients’ emergency room presentation PGx-guided pain management in Nigeria could potentially be of significance. The high frequency of IMs, largely driven by the *17 allele, aligns with known data showing *CYP2D6*17 is prevalent in African populations. ^30, 58, 59^ However, the PM frequency is lower than some previous reports from Nigeria, ^51^ which could reflect differences in genotyping platforms, while the presence of UMs resulting from gene duplications is also a critical finding. Notably, 9.3% of participants had an undetermined phenotype, a rate considerably higher than the typical less than 5% in general clinical settings for cases requiring copy number determination. ^60^ This high indeterminate rate is directly attributable to high genetic diversity within African populations and suggests the presence of novel or rare CYP2D6 alleles or haplotypes not captured by current standard genotyping panels and algorithms. ^27, 58^ Moreover, it also shows the extreme complexity of the CYP2D6 gene, which is a highly variable pharmacogene characterized by intricate structural variants (SVs) or copy number variants (CNVs) such as deletions, duplications, multiplications, and hybrid genes. ^60^ The instability of the CYP2D locus on chromosome 22, coupled with the presence of highly homologous pseudogenes (CYP2D7 and CYP2D8), significantly interferes with standard genetic analyses, making accurate haplotype assignment difficult. ^60^ Some complex haplotypes are even challenging to detect with standard microarray chips or short-read sequencing. ^61^ Accurate CYP2D6 phenotype prediction requires comprehensive testing for SVs/CNVs across multiple gene locations. ^61^ Thus, further investigation, potentially using sequencing, is needed to characterize these unknown variants and improve phenotype prediction accuracy. Phenotype prediction in the current study was performed by calculating the diplotype’s activity score and translating it to a metabolizer phenotype using the standardized guidelines published by CPIC.

The observed *CYP2D6* phenotype distribution has significant implications for pain management in Nigerian SCD patients, especially concerning commonly used opioids like codeine and tramadol. Both are prodrugs that rely on *CYP2D6* for conversion to their active metabolites (morphine and O-desmethyltramadol, respectively). Poor metabolizers on codeine therapy have shown through metabolic/pharmacokinetic and clinical endpoint studies to achieve a lower mean serum morphine AUC compared to normal metabolizers. ^62^ This results in inadequate pain control but could still potentially expose patients to adverse drug reactions from codeine. For both codeine and tramadol treatment failure in SCD-associated pain care has been significantly linked to CYP2D6 activity score, which was reported to be average in the IM range. ^63^ The occurrence of hypoanalgesic state with codeine or tramadol therapy could result in prolonged hospital admission, higher care costs and poor health-related QoL for SCD patients. ^63, 64^ Conversely, CYP2D6 UM status increases the risk of experiencing higher morphine levels by as much as 45% compared to CYP2D6 normal metabolizers for patients on codeine. ^65^ A similar phenomenon is observed with tramadol, and both could result in potentially fatal opioidergic effects. ^64^ These adverse drug effects occur in a spectrum of mild to severe. On the severe end of the spectrum, patients can experience sedation, dizziness, excessive nausea, vomiting, constipation, drug dependence and potentially fatal respiratory depression. ^66^ For both IM/PM, and UM the CPIC recommendation is to avoid codeine/tramadol use because of the potential for diminished effectiveness or serious toxicity, respectively. ^28^ Combining these categories, our findings suggest that at least 10.5% (UM + PM) of Nigerian SCD patients should not receive codeine or tramadol. Furthermore, another 26.0% (IM) may not achieve adequate pain relief or require careful titration. This means that over 36.6% of the SCD population in this region may experience either toxicity or lack of efficacy with these commonly prescribed analgesics if treatment is not guided by patients’ CYP2D6 status.

**Figure 4.**
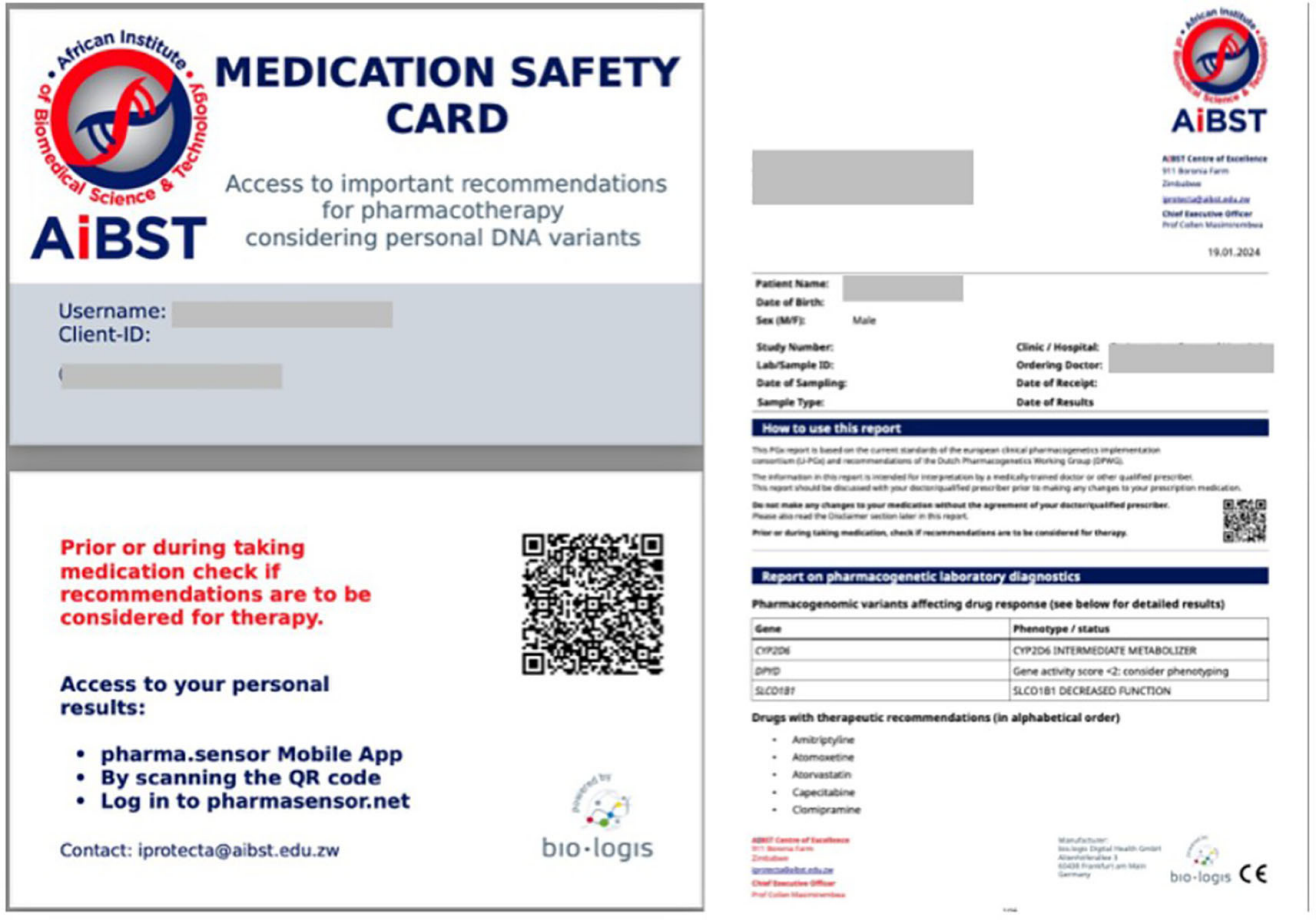
The medication safety card*. * This is a ‘credit card-like’ document that patient can carry with them and be able to access their results on the online GIMIS platform. Each patient will also have a hard copy of the results in case they do not have access to the internet. When visiting a doctor, the patient can carry their hard copy results or share their password with the doctor to see which medications patients should or should not take, and if there is a need for dose adjustments. For patients going to their reference clinic, the results will be in their local electronic patient data system.

A patient medication safety card ( Figure 4) was provided to each of the 503 SCD patients, intended for presentation at every clinical consultation to guide prescriptions, and detailed genotype and recommended treatment plan reports were given to each patient’s primary physician, building a database for future personalized pain management in Phase 2 of the study. This innovative safety card is a crucial step towards integrating pharmacogenomics (PGx) into routine clinical practice, particularly for managing VOCs in SCD, aligning with the role of clinical decision support (CDS) tools in preventing errors and improving health outcomes. ^67^ Similar to other patient-facing PGx tools with safety-code cards or QR codes, this card enhances patient-doctor communication by presenting complex PGx information in an accessible format. ^67^ Ultimately, this medication safety card represents a proactive and patient-centric approach, directly addressing VOC management by empowering both patients and doctors with personalized drug information, potentially reducing adverse drug reactions, improving treatment efficacy (especially for PGx-sensitive opioids in pain management), and bridging health literacy gaps, thereby enhancing patient safety in a resource-limited setting. ^28^

The study’s comprehensive genotyping and information dissemination efforts significantly position the cohort for a Phase 2 pharmacogenomics (PGx)-guided treatment trial, particularly through the pre-emptive genotyping of all 503 patients and the provision of detailed genotype reports and recommended treatment plans to primary physicians, aligning with the foundational assumption of proactive genotyping in CPIC guidelines. ^28^ While patient genotype databases have been established and detailed reports provided to healthcare providers, we have also addressed the crucial provider-related barriers in resource-limited settings through targeted education and capacity-building programs for the clinical team and other support staff to ensure effective PGx implementation. The availability of comprehensive CPIC guidelines with the team will support the feasibility of a PGx-guided pain management trial in the Phase 2 trial, which will pragmatically focus on optimizing existing, accessible opioid strategies given the cost and accessibility issues of newer SCD therapies in Africa. ^28^ Furthermore, the inherent variability across the six recruitment sites in terms of patient volume, genotype distribution, healthcare infrastructure, and resource availability has revealed the need for adaptive protocols and site-specific considerations in the Phase 2 trial design to ensure robust and generalizable findings. ^60, 67^

The phase 2 findings will be important in noting the successful integration of the PGx results, acceptability of the form of patient management to clinicians and sustainability of the model absence of electronic health records systems. Successful implementation and demonstrable sustainability could help provide evidence whereby SCD patients undergo pre-emptive PGx testing soon after birth and diagnosis of SCD.

## Conclusion

This study offers crucial insights into the feasibility and challenges of implementing pharmacogenomics (PGx)-guided treatment for Sickle Cell Disease (SCD) in high-burden, resource-limited settings like Nigeria. The cohort’s younger age and lower Body Mass Index (BMI) suggest a severe disease burden exacerbated by malnutrition and limited access to hydroxyurea, indicating that PGx interventions must be tailored to this distinct physiological context. Genotyping revealed the clinical utility of PGx, with a notable prevalence of CYP2D6 poor or intermediate metabolizer phenotypes suggesting potential for personalized opioid pain management, yet a 9% indeterminate rate for CYP2D6 genotyping presents a critical challenge due to the gene’s complexity and emphasizes the need for advanced methods and population-specific research. Readiness for a Phase 2 PGx-guided trial is bolstered by pre-emptive genotyping and the introduction of a PGx medication safety card, empowering patients and doctors in VOC management. The inherent variability across different sites also demands adaptive trial protocols and site-specific considerations for equitable implementation and generalizability. This study ultimately demonstrates the scientific promise of PGx while critically highlighting systemic, logistical, and human infrastructure challenges that must be addressed for equitable precision medicine in such settings. The pragmatic implementation study design employed here demonstrated the strength of a pan-African collaborative approach that is cost-effective without requirements for new laboratories to be set up at this stage in the study. It also provides a wider external validity to this regional approach to PGx implementation. This PGx implementation study has shown PGx acceptability in a Nigerian setting, the feasibility of clinical and laboratory coordination at a regional level and the ability to move to an effective decision-making input platform for a disease relevant to the Nigerian and greater sub-Saharan Africa population.

Moving forward, the long-term clinical utility and widespread adoption of this model will rely on integrating pre-emptive testing into national healthcare policies, which will require government subsidy and targeted training to ensure that genotyping costs become a sustainable investment in reducing adverse drug reactions and optimizing patient outcomes across Nigeria and the wider Sub-Saharan African region. Moreover, successful implementation of any PGx testing program will also include a comprehensive education of all stakeholders, particularly Primary Health Care Professionals (PHCPs). Training modules must be developed and integrated into continuing medical education to enhance PHCPs’ comprehension of PGx principles, their confidence in interpreting metabolizer phenotypes, and their ability to counsel patients effectively on genotype-guided prescribing decisions for critical medications like opioids.

## Ethics approval and consent to participate

All elements of the study were performed in accordance with the Declaration of Helsinki and the relevant regulations and laws governing research in Nigeria. Ethical approval for this study was obtained from the Ethics and Research Committee of Obafemi Awolowo University Teaching Hospitals Complex, Ile-Ife, with registration numbers International-IRB/IEC/0004553 and National-NHREC/17/03/2021. The ethical approval was received on 31 ^st^ July 2023 and the number assigned to this study is ERC/2022/07/17.

Written informed consent was obtained from all participants in a language the participant understood, which was English or the local language, mainly Yoruba and Igbo. Participants were thoroughly informed about the study’s purpose, procedures, potential risks and benefits, their right to withdraw at any time without penalty, and the confidentiality of their data. Ample opportunity was provided for participants to ask questions, and all queries were addressed to their satisfaction before consent was obtained. For participants who were minors (less than 18 years of age), written informed consent was obtained from their parents or legal guardians.

## Data Availability

Due to the highly sensitive nature of genetic data from human subjects, the datasets supporting this study are not publicly available to protect participant privacy and confidentiality. This restriction aligns with the ethical guidelines set forth by the Ethics Committee of Obafemi Awolowo University Teaching Hospitals Complex, Ile-Ife, which approved the study protocol, including its data access provisions. Qualified researchers, however, can apply for restricted access to de-identified data by submitting a research proposal, proof of their ethical approval, and signing a Data Access Agreement. All access requests should be directed to the corresponding authors ( badeagbo@oauife.edu.ng, cmasimirembwa@aibst.edu.zw) and will be reviewed by the study’s data governance committee to ensure ethical compliance.
